# Indirect tolerability comparison of Deutetrabenazine and Tetrabenazine for Huntington disease

**DOI:** 10.1186/s40734-017-0051-5

**Published:** 2017-03-01

**Authors:** Daniel O. Claassen, Benjamin Carroll, Lisa M. De Boer, Eric Wu, Rajeev Ayyagari, Sanjay Gandhi, David Stamler

**Affiliations:** 1 Vanderbilt University, 1161 21st Avenue South A-0118, Nashville, TN 37232 USA; 2 Teva Pharmaceuticals, Frazer, PA USA; 3 Teva Pharmaceuticals, La Jolla, CA USA; 4 Analysis Group, Inc., Boston, MA USA

**Keywords:** Deutetrabenazine, Tetrabenazine, Huntington disease, Indirect treatment comparison, Risk difference, Huntington’s chorea, Chorea, Safety, Tolerability, Movement disorders

## Abstract

**Background:**

Vesicular monoamine transporter 2 (VMAT2) inhibitors can improve hyperkinetic movements, and are effective treatment options for chorea of Huntington disease (HD). Tetrabenazine was assessed for treating chorea in the TETRA-HD trial, and while efficacious, there are tolerability concerns possibly due to its pharmacokinetic properties. Deutetrabenazine is a novel VMAT2 inhibitor that contains deuterium, which extends active metabolite half-lives and minimizes drug concentration fluctuations. In the First-HD trial, deutetrabenazine was efficacious in treating chorea and was generally well tolerated. In the absence of a head-to-head trial, we performed an indirect treatment comparison (ITC) of the tolerability of deutetrabenazine and tetrabenazine for the treatment of HD-associated chorea, as observed in the First-HD and TETRA-HD trials, using well-established comparison methods.

**Methods:**

Data from the Phase III, 12-week, parallel-group, clinical trials First-HD (*N* = 90) and TETRA-HD (*N* = 84) were used to conduct an ITC of the tolerability of deutetrabenazine versus tetrabenazine using two anchor-based methods: Bucher comparison for unadjusted ITCs, and matching indirect comparison for adjusted ITCs. Overall adverse events (AEs; mild, moderate, and severe), serious AEs, specific AEs occurring in ≥10% of patients, and discontinuations (all-cause and AE-related) were included in the analysis. The risk differences of these outcomes for deutetrabenazine and tetrabenazine were estimated by subtracting the applicable placebo-adjusted risk in First-HD from that of TETRA-HD. Sensitivity analyses were performed to address differences between trials, and *p*-values were obtained from z-tests.

**Results:**

Compared with tetrabenazine, deutetrabenazine was associated with a significantly lower risk of moderate to severe AEs and neuropsychiatric AEs including agitation, akathisia, depression, depression/agitated depression, drowsiness/somnolence, insomnia, and parkinsonism in both adjusted and unadjusted analyses (*p* < 0.05 for each). Deutetrabenazine had a significantly lower rate of dose reduction or dose reduction/suspension in the unadjusted and adjusted analyses (*p* < 0.001 for each). Deutetrabenazine resulted in numerically more mild AEs, such as diarrhea and coughing; however, these results were not statistically significant.

**Conclusions:**

This indirect treatment comparison demonstrates that for the treatment of HD chorea, deutetrabenazine has a favorable tolerability profile compared to tetrabenazine.

**Trial registration:**

ClinicalTrials.gov NCT01795859 and NCT00219804.

## Background

Huntington disease (HD) is a neurodegenerative disorder characterized by chorea and progressive motor, cognitive, and behavioral symptoms [1, 2]. The only US Food and Drug Administration (FDA)-approved drug for chorea associated with HD is tetrabenazine, a vesicular monoamine transporter 2 (VMAT2) inhibitor [3]. Tetrabenazine was evaluated in TETRA-HD, and while improving chorea, the adverse event (AE) profile raised tolerability concerns [3, 4].

Deutetrabenazine is a novel VMAT2 inhibitor structurally related to tetrabenazine, but it contains deuterium [5, 6], a naturally occurring, nontoxic form of hydrogen [7], that confers important metabolic advantages compared to tetrabenazine but does not change its target pharmacology. The introduction of deuterium in this compound attenuates drug metabolism and prolongs plasma half-life, resulting in more-uniform systemic exposure (i.e. reducing plasma fluctuations) [5, 6]. Compared with tetrabenazine, the differentiated pharmacokinetic properties have potential to improve the benefit-risk profile for patients. In First-HD, deutetrabenazine significantly reduced chorea and was generally well tolerated in patients with HD [5]. Incidences of most neuropsychiatric AEs were similar to or lower than those in the placebo group [5].

Deutetrabenazine and tetrabenazine have not been compared directly; however, because First-HD and TETRA-HD have very similar study designs and were performed by the same study consortium (the Huntington Study Group), we used well-established comparative effectiveness models [8, 9] to compare the tolerability profiles of the two medications before and after adjusting for any putative clinical differences in populations. In this analysis, AE differences were assessed before and after adjusting for cross-trial differences in baseline characteristics using the Bucher method for unadjusted analyses [8], and a matching-adjusted indirect comparison method for adjusted analyses. Given the AE profile observed in First-HD, we hypothesized that tolerability of deutetrabenazine was more favorable than that of tetrabenazine.

## Methods

### Trial comparability

**Fig. 1:**
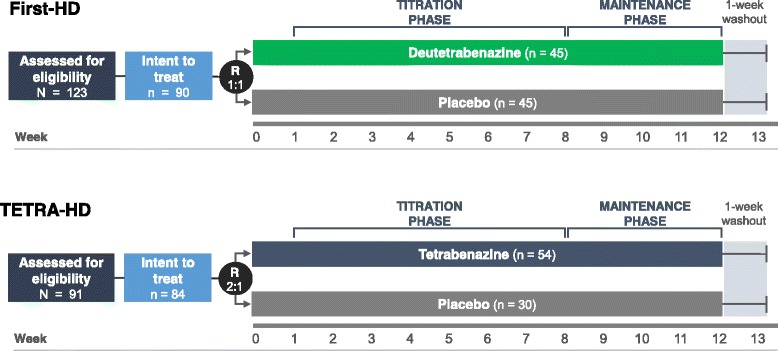
First-HD and TETRA-HD study designs. The First-HD and TETRA-HD study designs were very similar. For both studies, dose adjustments were allowed up to and including Week 7, followed by a maintenance period and a 1-week washout for both studies. R = randomization ratio

This was a retrospective study using previously reported clinical data. Full details of the First-HD (NCT01795859) and TETRA-HD (NCT00219804) study methods can be found in their respective primary reports [3, 5]. The Phase III First-HD and TETRA-HD trials were compared closely in terms of trial design, concomitant medication use, patients’ baseline characteristics, and definitions of safety outcomes. In both studies, patients underwent congruent titration periods, maintenance phases, during which dose was held stable, and 1-week washout periods (Fig. 1). The total treatment period in both studies was 12 weeks [3, 5]. Key First-HD inclusion criteria included manifest HD (CAG repeat length ≥37), ambulatory, total functional capacity (TFC) score ≥5, and TMC score ≥8 at screening and baseline [5]. In TETRA-HD, inclusion criteria included manifest HD (CAG repeat length ≥37), ambulatory, TFC score >5, and total maximal chorea (TMC) score ≥10 [3]. Exclusion criteria for both trials included disabling depression, dysphagia, or dysarthria [3, 5]. Stable treatment (for at least 8 weeks) with antidepressant medication was allowed in both trials [3, 5]. Prior use of tetrabenazine and current treatment with dopamine D2 receptor antagonists and drugs that prolong QT intervals were exclusionary in both trials (escitalopram and citalopram allowed in First-HD). There was a higher screen failure rate in First-HD, and this was driven by (a) ensuring patients were not taking tetrabenazine to avoid selecting patients with good tolerability to tetrabenazine (N.B. the First-HD protocol allowed patients who had received tetrabenazine but only if they discontinued at least 6 months prior to screening), (b) patients were excluded if the score on the Swallowing Disturbance Questionnaire was above a cut-off established for Parkinson’s disease [10], and (c) an independent functional capacity assessment was performed for all First-HD patients.

### Patients’ baseline characteristics

Baseline demographic and clinical differences between the First-HD and TETRA-HD cohorts were identified. Based on available sample sizes in each trial, we performed multivariable adjustments for up to three differing baseline characteristics between the two trials; age, TFC score, and TMC score. We considered these variables most clinically relevant for adjustment. Based on the review of these two cohorts, we determined that controlling for age, TFC score, and TMC was important given the potential that baseline cohort differences could impact medication tolerability, where advancing age [11], reduced TFC, and increased TMC may portend a more-advanced disease with increased likelihood of developing certain AEs.

### Indirect comparison

Tetrabenazine data were collected from the published clinical trial results and from the FDA-approved prescribing information [3, 12]. Aggregate data from TETRA-HD and patient-level data from First-HD were used to conduct an indirect comparison of AEs using anchor-based methodology. For unadjusted results, the Bucher method was used to compare two placebo-controlled trials with different comparators and to calculate a summary risk difference of the two treatments (i.e. tetrabenazine and deutetrabenazine) [8]. The Bucher method is a well-established and accepted analytical method used across disease states for conducting indirect comparisons [13–16].

A matching-adjusted indirect comparison was used to evaluate AE outcomes between patients treated with deutetrabenazine or tetrabenazine, which provided the ability to account for differences in the baseline characteristics of the two patient populations. This more sophisticated ITC method incorporates a propensity score model, which estimates the effect of a treatment, accounting for baseline clinical covariates that predict a treatment response. Thus, the propensity score of individual patient data from one trial is weighted such that the population resembles that of the comparator trial [9]. After this weighting adjustment, a Bucher-type comparison is then applied across the adjusted populations as a part of the matching-adjusted indirect comparison method. In this analysis, the First-HD patient baseline characteristics were weighted to resemble the TETRA-HD patients more closely.

Both methods provided estimates for the within-trial risk difference of deutetrabenazine and tetrabenazine for each safety outcome. Within-trial risk differences were calculated for each trial as the incidence of AEs in the active arm minus that in the placebo arm of the same trial. The summary risk differences between deutetrabenazine and tetrabenazine were estimated by subtracting the within-trial risk differences of TETRA-HD from the respective within-trial risk differences of First-HD. Number needed to harm (NNH) estimates were defined as the average number of patients that need to be treated with tetrabenazine instead of deutetrabenazine for an average of one additional patient to experience an AE. NNH estimates were calculated as the reciprocal of the summary risk differences.

### Safety outcomes

Any AE, moderate to severe AEs, mild AEs, serious AEs (SAEs), discontinuations (all-cause and AE-related), dose reductions, and specific AEs that occurred in ≥10% of patients were included in the analysis. An incidence rate of 10% was deemed clinically significant.

### Sensitivity analyses

In addition to the previously described analyses, a matching-adjusted indirect comparison was performed with adjustments for different combinations of baseline characteristics (age, TFC score, and TMC) to test the robustness of the findings. Also, AEs were coded according to Medical Dictionary for Regulatory Activities (MedDRA) and World Health Organization (WHO) Preferred Terms in First-HD and TETRA-HD, respectively. AEs reported in the tetrabenazine FDA-approved prescribing information were examined and compared with those reported in the primary TETRA-HD publication [3, 12]. Whenever ambiguity in definitions existed due to potential AE coding differences between the two trials, multiple versions of the First-HD AE definition were used within the analysis to assess the potential for any bias.

### Statistical analyses

Baseline characteristics and AEs from TETRA-HD [3] were summarized and compared to pre-existing summaries in the First-HD clinical study report. Baseline characteristics in the respective arms (i.e. active vs active and placebo vs placebo) for each trial were compared using chi-squared analysis for categorical variables and t-tests for continuous variables. *P*-values for risk differences were obtained using z-tests. Versions 9.2 and 9.3 of the SAS statistical software package were used for all analyses (SAS Institute, Inc.).

## Results

### Comparison of patients

While there were statistical differences between the overall populations enrolled in each clinical trial, the distributions for most variables were similar. Mean baseline TMC scores for patients enrolled in First-HD and TETRA-HD were 12.7 and 14.9, respectively [3]. Before adjustment, patients in the deutetrabenazine arm of First-HD had significantly higher ages, as well as TFC score, verbal fluency, symbol digit and Stroop word reading, behavioral assessment, functional checklist, independence scale and Barnes Akathisia Rating Scale (BARS) scores compared with the tetrabenazine arm of TETRA-HD (Table 1). In addition, patients in the deutetrabenazine arm in First-HD had significantly lower CAG repeat lengths as well as parkinsonism, TMS, and Unified Parkinson’s Disease Rating Scale (UPDRS) speech scores. The placebo arms in each trial only differed in gender, TMC score, and BARS score before weighting. These differences were further reduced after weighting.

**Table 1:** Comparison of baseline characteristics in First-HD versus TETRA-HD

	Before Matching				Before Matching^a^		After Matching				After Matching^a^	
	First-HD		TETRA-HD				First-HD		TETRA-HD			
Baseline Characteristics, Mean (SD)	DTB (*n* = 45)	PBO (*n* = 45)	TBZ (*n* = 54)	PBO (*n* = 30)	*p*-value Active	*p*-value PBO	DTB (*n* = 45)	PBO (*n* = 45)	TBZ (*n* = 54)	PBO (*n* = 30)	*p*-value Active	*p*-value PBO
Age	55.4 (10.3)	52.1 (13.4)	49.4 (12.4)	48.8 (10.5)	**0.009**	0.231	49.4 (14.3)	48.8 (12.3)	49.4 (12.4)	48.8 (10.5)	1.000	1.000
CAG repeat length	43.4 (2.7)	44.3 (4.4)	44.9 (3.4)	44.3 (3.7)	**0.013**	0.972	44.9 (4.1)	45.6 (4.6)	44.9 (3.4)	44.3 (3.7)	0.997	0.219
Gender, %												
Female	51.1	37.8	61.1	63.3	0.318	0.030	45.7	43.6	61.1	63.3	0.318	0.123
Male	48.9	62.2	38.9	36.7	0.318	0.030	54.3	56.4	38.9	36.7	0.318	0.123
Caucasian, %	100.0	84.4	92.6	96.7	0.062	0.093	100.0	80.0	92.6	96.7	**0.040**	0.035
UHDRS TMC^b^ (lower score indicates lower severity)	12.1 (2.7)	13.2 (3.5)	14.7 (3.8)	15.2 (4.4)	**<0.001**	0.041	14.7 (2.9)	15.2 (3.7)	14.7 (3.8)	15.2 (4.4)	1.000	1.000
UHDRS TMS^b^ (lower score indicates lower severity)	34.1 (13.2)	38.8 (15.2)	47.0 (16.7)	44.8 (15.5)	**<0.001**	0.099	41.4 (13.4)	46.7 (16.8)	47.0 (16.7)	44.8 (15.5)	0.201	0.677
Parkinsonism (lower score indicates lower severity)	9.3 (4.7)	11.7 (5.6)	13.8 (5.7)	12.8 (5.1)	**<0.001**	0.367	12.1 (5.3)	14.3 (6.0)	13.8 (5.7)	12.8 (5.1)	0.338	0.334
Gait (lower score indicates lower severity)	1.0 (0.7)	1.3 (0.7)	1.2 (0.6)	1.0 (0.5)	0.176	0.058	1.5 (0.6)	1.5 (0.7)	1.2 (0.6)	1.0 (0.5)	0.060	0.003
Verbal fluency (higher score reflects better cognitive ability)	25.2 (11.4)	23.3 (9.9)	18.9 (9.1)	18.7 (10.8)	**0.003**	0.064	20.9 (8.3)	21.6 (8.0)	18.9 (9.1)	18.7 (10.8)	0.312	0.209
Symbol digit (higher score reflects better cognitive ability)	23.3 (8.7)	23.4 (8.1)	18.1 (11.5)	24.4 (11.3)	**0.011**	0.689	23.2 (9.8)	20.8 (7.8)	18.1 (11.5)	24.4 (11.3)	0.097	0.140
Stroop test^c^ (higher score reflects better cognitive ability)												
Color naming	48.0 (16.9)	44.5 (13.9)	42.4 (14.3)	46.7 (16.4)	0.077	0.540	43.9 (15.2)	41.8 (11.7)	42.4 (14.3)	46.7 (16.4)	0.735	0.169
Word reading	65.9 (22.4)	55.7 (17.5)	53.8 (21.0)	56.3 (20.2)	**0.006**	0.896	63.1 (23.2)	52.7 (15.7)	53.8 (21.0)	56.3 (20.2)	0.224	0.419
Behavioral assessment (lower score indicates less impairment)	12.1 (12.4)	9.6 (10.1)	7.4 (7.3)	6.6 (6.2)	**0.025**	0.106	9.3 (10.5)	7.8 (9.5)	7.4 (7.3)	6.6 (6.2)	0.475	0.517
Functional checklist	21.6 (3.0)	20.8 (3.0)	18.8 (4.4)	19.6 (3.8)	**<0.001**	0.130	19.8 (3.4)	19.9 (3.0)	18.8 (4.4)	19.6 (3.8)	0.378	0.707
Independence scale (higher score indicates greater independence)	85.0 (9.4)	82.0 (9.3)	76.9 (11.6)	80.2 (9.4)	**<0.001**	0.414	80.3 (8.2)	79.3 (7.7)	76.9 (11.6)	80.2 (9.4)	0.172	0.670
Total functional capacity (higher scores indicate better capacity)	9.8 (2.3)	9.2 (2.0)	8.3 (2.4)	8.6 (2.3)	**0.001**	0.280	8.3 (2.0)	8.6 (1.8)	8.3 (2.4)	8.6 (2.3)	1.000	1.000
Epworth Sleepiness Scale (lower score indicates less daytime sleepiness)	4.6 (3.1)	5.4 (3.8)	3.4 (3.3)	4.4 (3.5)	0.073	0.220	6.2 (3.1)	4.8 (3.1)	3.4 (3.3)	4.4 (3.5)	**0.001**	0.614
Barnes Akathisia Rating Scale (higher scores indicate more akathisia and restlessness)	1.4 (1.6)	1.2 (1.9)	0.3 (0.7)	0.2 (0.6)	**<0.001**	0.001	0.8 (1.4)	1.0 (1.8)	0.3 (0.7)	0.2 (0.6)	0.079	0.020
UPDRS speech (lower score indicates less impairment)	0.7 (0.8)	1.0 (0.8)	1.3 (0.6)	1.1 (0.7)	**<0.001**	0.653	1.1 (0.7)	1.2 (0.7)	1.3 (0.6)	1.1 (0.7)	0.294	0.538

*DTB*, deutetrabenazine, *PBO* placebo, *SD* standard deviation, *TBZ* tetrabenazine, *TMC* total maximal chorea, *TMS* total motor score, *UHDRS* Unified Huntington’s Disease Rating Scale, *UPDRS* Unified Parkinson's Disease Rating Scale. Bolded values indicate significant differences between the active arms (*p* < 0.05)

^a^ *p*-values are for the comparison between the respective arms (i.e. active vs active and placebo vs placebo) of the First-HD and TETRA-HD trials

^b^In First-HD, baseline was defined as Day 0 visit; the definition was not available in TETRA-HD

^c^In First-HD, the Stroop interference score was reported as the difference between the predicted and raw color–word score; however, TETRA-HD did not provide a definition of the reported Stroop interference score. It is possible that either the raw or the predicted color–word score was reported in TETRA-HD; therefore, the Stroop interference score was not included in this table

### Comparison of safety outcomes

**Fig. 2:**
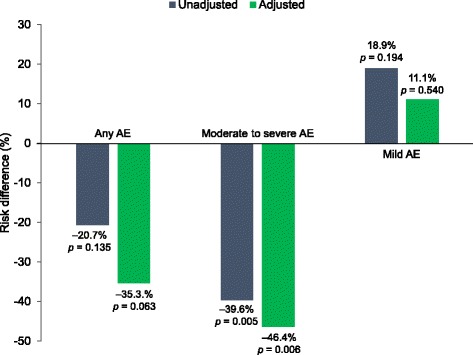
Risk differences for adverse events by severity. The risk differences were assessed before and after placebo-adjustment. Negative risk difference favors deutetrabenazine. *p*-values comparing the risk differences between deutetrabenazine and tetrabenazine were obtained from z-tests. AE = adverse event

Before and after adjustment, deutetrabenazine demonstrated significantly lower incidence rates for overall AEs and moderate to severe AEs than tetrabenazine (*p* < 0.001 for each; Table 2). This resulted in a significantly lower risk for moderate to severe AEs with deutetrabenazine compared with tetrabenazine in the unadjusted (−39.6%, 95% CI: −67.1, −12.2%; *p* = 0.005) and adjusted (−46.4%, 95% CI: −79.4, −13.3%; *p* = 0.006) analyses (Fig. 2, Table 3). Although not statistically significant, deutetrabenazine resulted in a greater risk of mild AEs in the unadjusted (18.9%, 95% CI: −9.6%, 47.4%; *p* = 0.194) and adjusted (11.1%, 95% CI: −24.4%, 46.6%; *p* = 0.540) analyses, specifically with a greater incidence of coughing (before adjustment *p* = 0.062, after adjustment *p* = 0.040) and diarrhea (before adjustment *p* = 0.788, after adjustment *p* = 0.951). Overall dose reductions and dose reductions/suspensions due to AEs occurred significantly less frequently with deutetrabenazine compared with tetrabenazine before and after placebo adjustment (*p* < 0.001 for all comparisons).

**Table 2:** Comparison of incidence of adverse events

Safety Outcome	Before Matching						After Matching					
	First-HD		TETRA-HD		*p*-value^a^ Active	*p*-value^a^ Placebo	First-HD		TETRA-HD		*p*-value^a^ Active	*p*-value^a^ Placebo
	DTB (*n* = 45)	PBO (*n* = 45)	TBZ (*n* = 54)	PBO (*n* = 30)			DTB (*n* = 45)	PBO (*n* = 45)	TBZ (*n* = 54)	PBO (*n* = 30)		
Any AE, %	60.0	60.0	90.7	70.0	**<0.001**	0.377	41.2	55.8	90.7	70.0	**<0.001**	0.253
Moderate to severe	22.2	26.7	68.5	33.3	**<0.001**	0.534	15.7	26.9	68.5	33.3	**<0.001**	0.584
Mild	37.8	33.3	22.2	36.7	0.090	0.766	25.5	28.8	22.2	36.7	0.759	0.503
At least one SAE, %	2.2	2.2	7.4	0.0	0.241	0.411	0.6	1.4	7.4	0.0	0.059	0.613
Discontinuation for any reason, %	2.2	4.4	9.3	3.3	0.144	0.810	0.6	4.8	9.3	3.3	**0.030**	0.760
Discontinuation due to AE, %	2.2	2.2	9.3	0.0	0.144	0.411	0.6	1.7	9.3	0.0	**0.030**	0.555
Dose reduction due to AE, %	6.7	6.7	44.4	3.3	**<0.001**	0.529	6.3	5.6	44.4	3.3	**<0.001**	0.624
Dose reduction/suspension due to AE, %	8.9	8.9	44.4	3.3	**<0.001**	0.345	6.8	7.3	44.4	3.3	**<0.001**	0.420
Individual AEs^b^												
Agitation, %	2.2	0.0	14.8	0.0	**0.030**	–	0.6	0.0	14.8	0.0	**0.004**	–
Akathisia (PI), %	2.2	2.2	18.5	0.0	**0.010**	0.411	1.4	1.8	18.5	0.0	**0.002**	0.545
Anxiety, %	2.2	2.2	14.8	3.3	**0.030**	0.770	0.8	1.4	14.8	3.3	**0.004**	0.577
Coughing, %	0.0	0.0	7.4	10.0	0.062	0.030	0.0	0.0	7.4	10.0	**0.040**	0.071
Depression, %	2.2	6.7	14.8	0.0	**0.030**	0.149	0.2	6.2	14.8	0.0	**0.003**	0.202
Depression/agitated depression, %	4.4	6.7	14.8	0.0	0.088	0.149	0.8	6.2	14.8	0.0	**0.004**	0.202
Diarrhea, %	8.9	0.0	7.4	10.0	0.788	0.030	7.1	0	7.4	10.0	0.951	0.071
Drowsiness/somnolence, %	11.1	4.4	31.5	3.3	**0.015**	0.810	8.9	3.6	31.5	3.3	**0.006**	0.947
Fall, %	4.4	8.9	16.7	13.3	0.054	0.541	3.3	10.5	16.7	13.3	**0.021**	0.748
Fatigue, %	6.7	4.4	22.2	13.3	**0.032**	0.164	3.3	2.7	22.2	13.3	**0.002**	0.108
Insomnia, %	6.7	4.4	25.9	0.0	**0.011**	0.242	3.9	2.2	25.9	0.0	**<0.001**	0.456
Nausea, %	2.2	4.4	13.0	6.7	0.051	0.675	0.6	4.0	13.0	6.7	**0.007**	0.641
Parkinsonism (PI), %	0.0	0.0	14.8	0.0	**0.007**	–	0.0	0.0	14.8	0.0	**0.002**	–
Vomiting, %	0.0	6.7	5.6	3.3	0.108	0.529	0.0	6.4	5.6	3.3	0.080	0.548

*AE* adverse event, *DTB* deutetrabenazine, *PBO* placebo, *SAE* serious adverse event, *TBZ* tetrabenazine

^a^ *p*-values are for the comparison between respective arms (i.e. active vs active and placebo vs placebo) for the First-HD and TETRA-HD trials. Bolded values indicate significant differences between the active arms (*p* < 0.05). ^b^Those safety outcomes sourced from the tetrabenazine FDA-approved prescribing information have been identified by “(PI)”

**Table 3:** Summary risk difference comparison of adverse events

Safety Outcome	Unadjusted Risk Difference			Adjusted Risk Difference		
	DTB vs TBZ	95% CI	*p*-value	DTB vs TBZ	95% CI	*p*-value
Any AE, %	−20.7	−47.9, 6.4	0.135	−35.3	−72.4, 1.8	0.063
Moderate to severe	**−39.6**	**−67.1, −12.2**	**0.005**	**−46.4**	**−79.4, −13.3**	**0.006**
Mild	18.9	−9.6, 47.4	0.194	11.1	−24.4, 46.6	0.540
At least one SAE, %	−7.4	−16.7, 1.9	0.117	−8.2	−17.4, 1.0	0.080
Discontinuation for any reason, %	−8.1	−20.6, 4.3	0.201	−10.1	−23.4, 3.2	0.136
Discontinuation due to AE, %	−9.3	−19.1, 0.6	0.065	**−10.4**	**−20.3, −0.4**	**0.041**
Dose reduction due to AE	**−41.1**	**−59.1, −23.1**	**<0.001**	**−40.5**	**−62.0, −19.0**	**<0.001**
Dose reduction/suspension due to AE	**−41.1**	**−60.0, −22.3**	**<0.001**	**−41.6**	**−63.9, −19.3**	**<0.001**
Individual AEs^a^						
Agitation, %	**−12.6**	**−23.0, −2.2**	**0.018**	**−14.2**	**−24.6, −3.8**	**0.007**
Akathisia (PI), %	**−18.5**	**−30.5, −6.5**	**0.003**	**−18.9**	**−32.0, −5.8**	**0.005**
Anxiety, %	−11.5	−24.4, 1.5	0.083	−12.0	−25.2, 1.2	0.074
Coughing, %	2.6	−10.2, 15.4	0.692	2.6	−10.2, 15.4	0.692
Depression, %	**−19.3**	**−32.0, −6.6**	**0.003**	**−20.8**	**−33.8, −7.8**	**0.002**
Depression/agitated depression, %	**−17.0**	**−30.4, −3.7**	**0.013**	**−20.2**	**−33.9, −6.5**	**0.004**
Diarrhea, %	11.5	−3.8, 26.8	0.141	9.7	−9.4, 28.7	0.320
Drowsiness/somnolence, %	**−21.5**	**−39.2, −3.7**	**0.018**	**−22.9**	**−44.9, −0.8**	**0.042**
Fall, %	−7.8	−26.5, 11.0	0.417	−10.6	−32.1, 10.9	0.336
Fatigue, %	−6.7	−25.6, 12.3	0.491	−8.3	−28.3, 11.7	0.416
Insomnia, %	**−23.7**	**−38.7, −8.7**	**0.002**	**−24.3**	**−40.9, −7.6**	**0.004**
Nausea, %	−8.5	−23.2, 6.1	0.255	−9.7	−24.7, 5.4	0.207
Parkinsonism (PI), %	**−14.8**	**−24.3, −5.3**	**0.002**	**−14.8**	**−24.3, −5.3**	**0.002**
Vomiting, %	−8.9	−20.4, 2.6	0.129	−8.7	−21.1, 3.8	0.173

*AE* adverse event, *CI* confidence interval, *DTB* deutetrabenazine, *SAE* serious adverse event, *TBZ* tetrabenazine

^a^Those safety outcomes sourced from the tetrabenazine FDA-approved prescribing information have been identified by “(PI)”. Bolded values indicate significant risk differences between deutetrabenazine and tetrabenazine (*p* < 0.05)

**Fig. 3:**
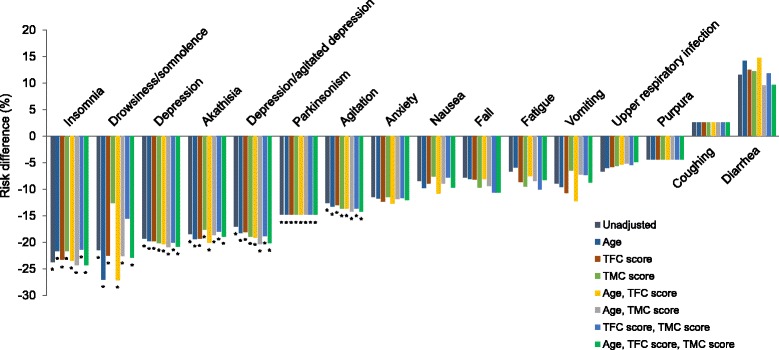
Risk difference for specific adverse events. The risk differences for specific adverse events, including insomnia, drowsiness/somnolence, depression, akathisia, depression/agitated depression, parkinsonism, agitation, anxiety, vomiting, nausea, fall, fatigue, vomiting, upper respiratory infection, purpura, diarrhea, and coughing were assessed. Negative risk difference favors deutetrabenazine. The figure presents unadjusted data, in addition to data adjusted by baseline characteristics (TMC, TFC, and/or age). *p < 0.05. *p*-values comparing the risk differences between deutetrabenazine and tetrabenazine were obtained from z-tests. TFC = total functional capacity, TMC = total maximal chorea

Deutetrabenazine demonstrated a significantly lower incidence of several individual AEs compared with tetrabenazine (Table 2). Before adjustment, patients treated with deutetrabenazine had significantly lower incidence of agitation, akathisia, anxiety, depression, drowsiness/somnolence, fatigue, insomnia, and parkinsonism compared with those treated with tetrabenazine (*p* < 0.05 for each). After adjustment, in addition to the AEs mentioned above, deutetrabenazine demonstrated significantly lower incidences of depression/agitated depression, falls, and nausea (*p* < 0.05 for each). Before adjustment, there was significantly lower risk for agitation (−12.6%, 95% CI: −23.0, −2.2%; *p* = 0.018), akathisia (−18.5%, 95% CI: −30.5, −6.5%; *p* = 0.003), depression (−19.3%, 95% CI: −32.0, −6.6%; *p* = 0.003), depression/agitated depression (−17.0%, 95% CI: −30.4, −3.7%; *p* = 0.013), drowsiness/somnolence (−21.5%, 95% CI: −39.2, −3.7%; *p* = 0.018), insomnia (−23.7%, 95% CI: −38.7, −8.7%; *p* = 0.002), and parkinsonism (−14.8%, 95% CI: −24.3, −5.3%; *p* = 0.002) with deutetrabenazine treatment compared with tetrabenazine treatment (Fig. 3, Table 3). After adjustment, the risk for the following AEs remained significantly lower for deutetrabenazine compared with tetrabenazine: agitation (−14.2%, 95% CI: −24.6, −3.8%; *p* = 0.007), akathisia (−18.9%, 95% CI: −32.0, −5.8%; *p* = 0.005), depression (−20.8, 95% CI: −33.8, −7.8%; *p* = 0.002), depression/agitated depression (−20.2%, 95% CI: −33.9, −6.5%; *p* = 0.004), drowsiness/somnolence (−22.9%, 95% CI: −44.9, −0.8%; *p* = 0.042), insomnia (−24.3%, 95% CI: −40.9, −7.6; *p* = 0.004), parkinsonism (−14.8%, 95% CI: −24.3, −5.3%; *p* = 0.002). The risk for other AEs did not significantly differ between treatment groups; however, deutetrabenazine was associated with numerically lower risk of most of these AEs compared with tetrabenazine, except for coughing and diarrhea. To test whether there was any effect of multiple comparisons on the statistically significant individual AE findings, we performed the Benjamini-Hochberg false discovery rate controlling procedure at the 0.1 level. The statistical significance of the results remained unchanged in both the unadjusted and adjusted analyses.

**Fig. 4:**
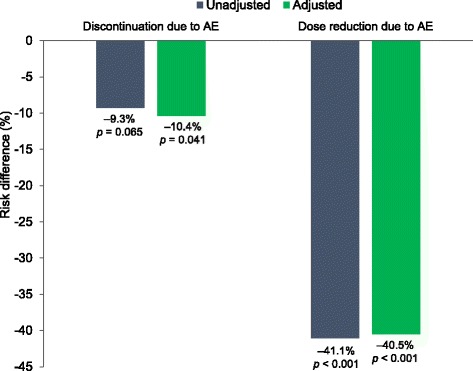
Risk differences for discontinuations and dose reductions due to adverse events. The risk differences were assessed before and after placebo-adjustment. Negative risk difference favors deutetrabenazine. *p*-values comparing the risk differences between deutetrabenazine and tetrabenazine were obtained from z-tests. AE = adverse event

Deutetrabenazine-treated patients had significantly lower risk for dose reduction or dose reduction/suspension compared with those treated with tetrabenazine in the unadjusted (−41.1%, 95% CI: −59.1, −23.1%; *p* < 0.001 for reduction and −41.1%, 95% CI: −60.0, −22.3%; *p* < 0.001 for reduction/suspension) and adjusted (−40.5%, 95% CI: −62.0, −19.0%; *p* < 0.001 for dose reduction and −41.6%, 95% CI: −63.9, −19.3%; *p* < 0.001 for dose reduction/suspension) analyses (Fig. 4, Table 3). The incidence of discontinuations due to AEs was numerically lower with deutetrabenazine versus tetrabenazine in the unadjusted analysis (*p* = 0.144), and was significantly lower with deutetrabenazine compared with tetrabenazine in the adjusted analysis (*p* = 0.030). While there was a trend toward lower risk for discontinuations due to AEs with deutetrabenazine compared with tetrabenazine before adjustment (−9.3%, 95% CI: −19.1, 0.6%; *p* = 0.065), discontinuations due to AEs in deutetrabenazine patients were significantly lower after adjustment (−10.4%, 95% CI: −20.3, −0.4%; *p* = 0.041) (Fig. 4, Table 3).

NNH values were statistically significantly favorable for deutetrabenazine compared with tetrabenazine for moderate to severe AEs in the unadjusted analysis (NNH = 3, 95% CI: 1, 8), and adjusted analysis (NNH = 2, 95% CI: 1, 7). That is, after adjustment, if two patients were treated with tetrabenazine instead of deutetrabenazine, one more patient, on average, would experience a moderate to severe AE. Likewise, NNH were significantly favorable for deutetrabenazine compared with tetrabenazine for the following adverse events in both adjusted and unadjusted analyses: agitation (NNH = 8 [95% CI: 4, 46] unadjusted; NNH = 7 [95% CI: 4, 26] adjusted); akathisia (NNH = 5 [95% CI: 3, 15] unadjusted; NNH = 5 [95% CI: 3, 17] adjusted); depression (NNH = 5 [95% CI: 3, 15] unadjusted; NNH = 5 [95% CI: 3, 13] adjusted); depression/agitated depression (NNH = 6 [95% CI: 3, 27] unadjusted; NNH = 5 [95% CI: 3, 15] adjusted); drowsiness/somnolence (NNH = 5 [95% CI: 3, 27] unadjusted; NNH = 4 [95% CI: 2, 119] adjusted); insomnia (NNH = 4 [95% CI: 3, 12] unadjusted; NNH = 4 [95% CI: 2, 13] adjusted); and parkinsonism (NNH = 7 [95% CI: 4, 19] for both). No other AEs analyzed were statistically significant.

### Sensitivity analyses

There were no meaningful changes in the results with different combinations of baseline characteristics included for adjustments in the matching-adjusted indirect comparisons (Fig. 3). Results based on the tetrabenazine FDA approved prescribing information were similar to those based on the TETRA-HD publication [3, 12]. Results for depression and dose reduction due to AEs were not sensitive to different definitions used by each trial (Table 3).

## Discussion

Results of this comparative analysis emphasize that compared to tetrabenazine, patients treated with deutetrabenazine experienced a significantly lower risk for moderate to severe AEs and dose reductions due to AEs in the unadjusted and adjusted data sets. Discontinuations were also significantly lower in the adjusted data set. These results corroborate the expected improvement in the benefit-risk profile of deutetrabenazine, potentially due to the differentiated pharmacokinetic profile resulting from deuterium substitution and attenuated metabolism. This is particularly relevant because long-term evaluation of tetrabenazine use in patients with HD indicates that tetrabenazine tolerability, rather than a plateau of efficacy, is often dose-limiting [17].

The European Huntington’s Disease Network (EHDN) REGISTRY study reported that <10% of patients with HD taking medications receive tetrabenazine [18]. Clinical management of chorea is not uniform, and comorbid psychiatric comorbidities commonly seen in HD patients (e.g. delusions, behavioral outbursts, and psychosis) often necessitate the use of neuroleptics, which can also provide improvements to choreiform movements. Of course, neuroleptics also have significant side effects, such as worsening parkinsonism, metabolic derangements (among other side effects), and poor tolerability which can limit their use. However, given our ITC findings, if improved tolerability of deutetrabenazine is replicated in larger HD populations, this would potentially allow for increased options for managing chorea and titration up to higher doses, thus maximizing chorea control with fewer dose-limiting AEs [19].

We note that deutetrabenazine had a significantly lower risk for several neuropsychiatric AEs compared with tetrabenazine, including insomnia, drowsiness/somnolence, depression, akathisia, depression/agitated depression, parkinsonism, and agitation. The risk difference for anxiety and fatigue also favored deutetrabenazine, although these did not reach significance. All of the seven AEs, agitation, akathisia, depression, depression/agitated depression, drowsiness/somnolence, insomnia, and parkinsonism, remained significantly lower with deutetrabenazine treatment even after applying Benjamini-Hochberg corrections. This further highlights the robustness of these results. In HD, disease severity is often associated with the progression of psychiatric co-morbidities, which are likely secondary to the disease process [20]. Although tetrabenazine is the only FDA-approved drug for chorea associated with HD, there is concern regarding exacerbation of neuropsychiatric comorbidities (especially depression) [21], frequently encountered in patients with HD; however, treated depression is not a contraindication for its use [21, 22]. Approximately 40% of patients with HD report significant depressive symptoms, such as low self-esteem, sadness, anxiety, and suicidal ideation [23]. The most recent American Academy of Neurology guidelines name depression and parkinsonism as the most concerning AEs related to tetrabenazine, and close monitoring of all patients is recommended [22]; depression AEs have been shown to occur in patients with and without pre-existing depression [21]. The significantly lower risk for neuropsychiatric AEs with deutetrabenazine as compared with tetrabenazine, including depression and parkinsonism, suggests that deutetrabenazine offers patients an alternative treatment option that may be less likely to induce or exacerbate neuropsychiatric symptoms. The lower risks of AEs observed with deutetrabenazine may be due to the substitution of hydrogen with deuterium at specific sites, which attenuates the metabolism and results in a more stable pharmacokinetic profile.

There are potential limitations of this analysis that should be considered. Before adjustment, patients in the deutetrabenazine arm in First-HD had higher TFC scores and lower CAG repeat lengths and TMS scores, suggesting that patients treated with deutetrabenazine were less advanced in their disease than those treated with tetrabenazine in TETRA-HD, and may have a different disease course. We suspect that lower CAG repeat length in the First-HD group was driven primarily by the recruitment of tetrabenazine-naïve patients, and less likely by the strict swallowing and functional capacity assessment criteria in the study. The analysis adjusted for important clinical differences between the First-HD and TETRA-HD patient groups. However, the sample sizes precluded all clinical and demographic variables to be included in the model. So while we were able to adjust for key clinical differences (TFC, age, and TMC), we were not able to adjust for some baseline characteristics, such as CAG repeat length and TMS scores, due to sample size and model parsimony considerations. The criteria used to select which variables were to be included in the model included collinearity with other variables to achieve a robust adjustment of differences between study groups. As with any indirect comparison, the matching-adjusted indirect comparison method cannot adjust for differences in patient characteristics that were not observed, or not reported in one of the trials. While AE definitions were generally analogous, they could be compared directly only when the TETRA-HD publication reported the definition. In addition, sample sizes in the trials only permitted adjustment for up to three population characteristics at a time; however, the results remained largely unchanged regardless of characteristics included for adjustment.

## Conclusions

In both adjusted and unadjusted comparisons, deutetrabenazine demonstrated significantly lower rates of both aggregated and individual safety outcomes compared with tetrabenazine. This is especially important in patients with HD, who may have a high disease burden and several neuropsychiatric comorbidities. First-HD and TETRA-HD had similar inclusion/exclusion criteria, protocols, patient populations, study endpoints, and interventions, and therefore required limited adjustment for matching baseline data. Moreover, the clinical sites were similar in experience and training. Because of this, the outcomes were consistent across sensitivity analyses even after modifying the definitions of AEs, data sources (primary publication vs FDA-approved prescribing information), and adjustments for different baseline characteristics. These results reinforce the favorable safety profile observed during the First-HD study, which may be attributed to the differentiated pharmacokinetic profile of deutetrabenazine resulting from deuterium substitution. Taken together, these data suggest that deutetrabenazine may be a well-tolerated treatment option for patients with chorea associated with HD, and may allow titration to an optimal therapeutic dose with fewer treatment disruptions due to AEs. The potential benefit of this optimized therapy is that greater efficacy, i.e., chorea control, may be achieved which may in turn lead to improved function [5]. Future clinical trials of deutetrabenazine are needed to confirm long-term clinical benefits of deutetrabenazine and patient persistence on treatment. In addition, a head to head trial would provide the best evidence for comparison of tolerability between the two medications.

## Abbreviations

AE: Adverse event
BARS: Barnes Akathisia Rating Scale
CI: Confidence interval
EHDN: European Huntington’s Disease Network
FDA: US Food and Drug Administration
HD: Huntington disease
MedDRA: Medical Dictionary for Regulatory Activities
NNH: Number needed to harm
SAE: Serious adverse event
TFC: Total functional capacity
TMC: Total maximal chorea
TMS: Total motor score
UHDRS: Unified Huntington’s Disease Rating Scale
UPDRS: Unified Parkinson’s Disease Rating Scale
VMAT2: Vesicular monoamine transporter 2
WHO: World Health Organization
